# Fostemsavir analog BMS-818251 has enhanced viral neutralization potency and similar escape mutation profile

**DOI:** 10.1128/aac.01910-24

**Published:** 2025-08-27

**Authors:** Yen-Ting Lai, Adam S. Dingens, Megan DeMouth, Sabrina Helmold Hait, Sijy O'Dell, Arne Schon, Adam S. Olia, Tao Wang, Hannah R. Shrader, Sarah E. Lovelace, Amarendra Pegu, Nicole A. Doria-Rose, John R. Mascola, Jesse D. Bloom, Peter D. Kwong

**Affiliations:** 1 Vaccine Research Center, National Institute of Allergy and Infectious Diseases, National Institutes of Health 35037, Bethesda, Maryland, USA; 2 N-MER Therapeutics, Boston, Massachusetts, USA; 3 Basic Sciences Division and Computational Biology Program, Fred Hutchinson Cancer Research Center 7286 , Seattle, Washington, USA; 4 Howard Hughes Medical Institute, Seattle, Washington, USA; 5 Department of Biology, Johns Hopkins University 1466, Baltimore, Maryland, USA; 6 Department of Discovery Chemistry, Bristol-Myers Squibb Research and Development, Cambridge, Massachusetts, USA; 7 Department of Molecular Technologies, Bristol-Myers Squibb Research and Development, Cambridge, Massachusetts, USA; 8 Aaron Diamond AIDS Research Center, Vagelos College of Physicians and Surgeons, Columbia University 5798, New York, New York, USA; 9 Department of Biochemistry and Molecular Biophysics, Columbia University, New York, New York, USA; IrsiCaixa Institut de Recerca de la Sida, Barcelona, Spain

**Keywords:** HIV-1, entry inhibitor, fostemsavir, BMS-818251, deep mutational scanning, resistance, *ex vivo* neutralization

## Abstract

BMS-818251, a fostemsavir analog, is a next-generation HIV-1 attachment inhibitor with enhanced potency and a similar resistance profile. By using *ex vivo* viral outgrowth assays with HIV+ donor samples, we demonstrate here that BMS-818251 exhibits superior viral suppression compared to temsavir, the active form of fostemsavir. To map potential resistance pathways, we employed deep mutational scanning and pseudotyped virus neutralization assays to identify escape mutations within the HIV-1 envelope glycoprotein (Env). These mutations were largely clustered around the BMS-818251 binding site, with key resistance mutations reducing drug-binding affinity. Several of the enriched mutations, such as S375I/N, M426L, and M475I, have been previously observed in fostemsavir-treated patients, highlighting their clinical relevance. Isothermal titration calorimetry revealed reduced binding affinity as the primary mechanism of resistance, though with notable exceptions, such as R429G, suggesting additional factors to influence viral escape. *Ex vivo* Env sequencing confirmed selection of resistance mutations under BMS-818251 pressure, reinforcing the predictive value of deep mutational scanning for *in vivo* resistance monitoring. Compared to fostemsavir, BMS-818251 achieved more effective viral suppression at lower concentrations, even in donor samples harboring preexisting resistance mutations. These findings support the continued development of BMS-818251 as a promising alternative to fostemsavir, with potential benefits for patients with multidrug-resistant HIV-1.

## INTRODUCTION

The HIV-1 attachment inhibitor fostemsavir (brand name Rukobia), approved by the U.S. Food and Drug Administration in 2020, treats multidrug-resistant HIV-1 in heavily treatment-experienced patients (1). Unlike other antiretroviral drugs that target reverse transcription, integration, or protease activity, fostemsavir prevents viral entry by binding to the HIV-1 envelope glycoprotein (Env) gp120, locking it in a prefusion state and blocking its interaction with the CD4 receptor. Because of its unique mechanism of action, fostemsavir exhibits no cross-resistance with other classes of HIV-1 entry inhibitors, such as maraviroc (a CCR5 antagonist) or enfuvirtide (a fusion inhibitor) (2–4). However, clinical studies (5, 6) and post-approval clinical uses (7, 8) have shown that resistance mutations can emerge in patients undergoing fostemsavir monotherapy, potentially limiting its long-term efficacy.

BMS-818251 is a next-generation analog of fostemsavir designed to enhance binding affinity to gp120 and to improve antiviral potency. Previous *in vitro* studies have demonstrated that BMS-818251 exhibits a more than 10-fold increase in neutralization potency compared to fostemsavir across a diverse panel of 208 HIV-1 strains (9). Despite its promising activity, the potential for resistance development remains a key consideration. Given that fostemsavir-resistant variants have been detected in clinical settings, understanding how HIV-1 escapes inhibition by BMS-818251 is critical for assessing its therapeutic viability.

To characterize the resistance profile of BMS-818251, we employed a comprehensive deep mutational scanning approach to map systematically escape mutations across the HIV-1 envelope glycoprotein. This method enables high-resolution identification of amino acid substitutions that reduce drug susceptibility. We complemented these findings with pseudotyped virus neutralization assays to validate the functional impact of selected resistance mutations. Furthermore, we investigated the biophysical mechanisms underlying resistance using isothermal titration calorimetry (ITC) to measure the binding affinity of recombinant Env mutants to BMS-818251.

Beyond *in vitro* analyses, we evaluated the *ex vivo* potency of BMS-818251 using viral outgrowth assays with HIV+ donor-derived CD4^+^ T cells. This approach allowed us to assess the ability of BMS-818251 to suppress viral replication in a physiologically relevant setting. By sequencing Env from these *ex vivo* cultures, we identified resistance mutations that emerged under drug pressure, providing insights into clinically relevant escape pathways.

Our findings reveal that BMS-818251 maintains a similar but slightly expanded resistance profile compared to fostemsavir, with key mutations clustered around the drug-binding site on gp120. The primary mechanism of resistance appears to be reduced binding affinity, although certain mutations, such as R429G, suggest alternative escape mechanisms. Importantly, BMS-818251 demonstrated superior suppression of viral replication in *ex vivo* assays, supporting its continued development as a next-generation HIV-1 attachment inhibitor. These results underscore the importance of resistance monitoring and highlight the potential clinical benefits of BMS-818251 in overcoming limitations associated with fostemsavir.

## RESULTS

**Fig 1:**
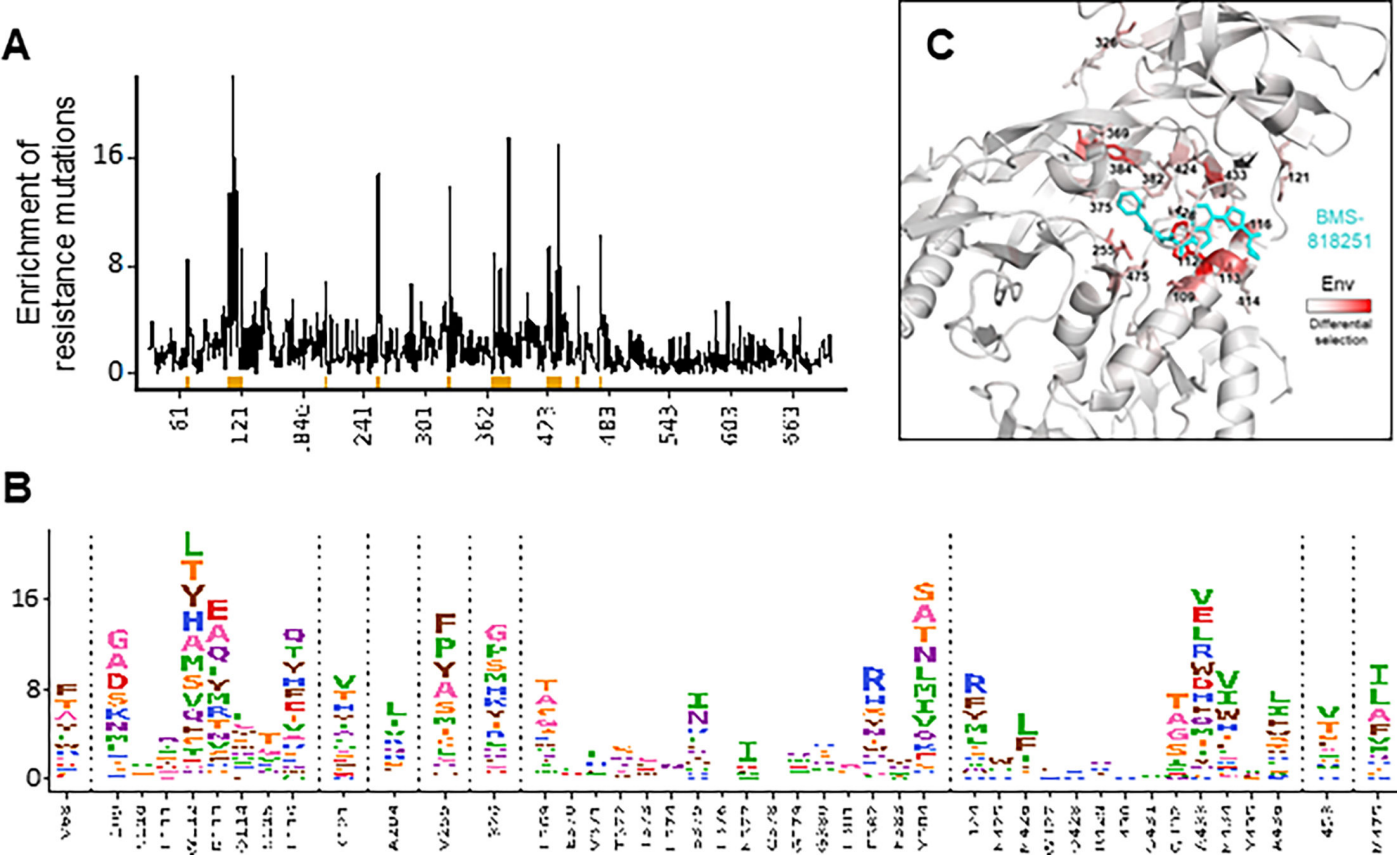
A complete map of BMS-818251 single-amino acid resistance mutations on BG505 Env (**A**) The positive site differential selection is plotted across the mutagenized portion of BG505 Env; the height of the line corresponds to the log of the enrichment of all mutations at each site. (**B**) The mutation-level resistance profile for regions of strong enrichment that are underlined in orange in panel **A**. The height of each amino acid is proportional to its differential selection, which is the logarithm of the relative enrichment of that mutation in the BMS-818251-selected condition relative to the non-selected control. Non-contiguous residues are divided by vertical dotted lines. Amino acid identities are color-coded based on their biochemical properties (small and non-polar amino acids [A and G] shown as pink; polar amino acids [S, T, and C] shown as orange; neutral [Q and N] shown as purple; basic [K, R, and H] shown as blue; acidic [D and E] shown as red; aromatic [F, Y, and W] shown as brown; and remaining hydrophobic amino acids [V, L, I, P, and M] shown as green). See Fig. S1, File S1, for all mutation-level values. (**C**) The positive site differential selection is mapped (from white to red) onto a monomer of the Env crystal structure bound by BMS-818251 (PDB code: 6MU7). Amino acids with significant enrichment in the binding interface are shown with sticks to highlight details.

To comprehensively identify Env mutations that may confer resistance to BMS-818251, we first utilized a deep mutational scanning-based approach to quantify the effect of all single-amino acid mutations to BG505 Env on BMS-818251 resistance (10). By comparing the BMS-818251-selected and non-selected viral libraries, we observed that enrichment of potential resistance mutations occurred at a number of regions, including sites I109, W112, D113, L116, K121, V255, I326, L369, S375, F382, Y384, I424, A433, M434, and M475 (Fig. 1A and B; Fig. S1). While these sites of enrichment were discontinuous in sequence space, they were remarkably well clustered around the BMS-818251 binding site (9) (Fig. 1C), with the exception of K121, I326, and L369 (Fig. 1C). Mutations that had previously been reported to affect resistance to fostemsavir in clinical trials (S375H/I/N/M/T, M426L/P, M434I/K, and M475I) (5) were also enriched in this assay. It is noteworthy that many of these mutations that arose during treatment *in vivo* were among the strongest enriched mutations at their respective sites (e.g., S375I/N, M426L, and M475I) in the deep mutational scanning assay. Additionally, this data set revealed many more possible *in vitro* escape mutations compared to those previously observed.

To further evaluate the impact of HIV-1 envelope mutations on the resistance to BMS-818251, we used pseudovirus neutralization assays to determine the effects of select resistance mutations identified by deep mutational scanning or structural analysis. Eleven single mutations—W112L, D113E, V255F, S375I, F382R, M426L, R429G, Q432T, A433V, M434I, and M475I—were introduced individually onto the envelope protein of the BG505 pseudoviruses. V255F abolished the entry of pseudovirus into reporter cells, while the other 10 mutations led to pseudoviruses with normal entry. Among the 10 mutants that can infect reporter cells, 4 mutants—W112L, D113E, F382R, and M426L—became fully resistant to BMS-818251 (IC_50_ increased by more than 125-fold when compared to wild type) (Table 1). The other five mutants—S375I, Q432T, A433V, M434I, and M475I—became resistant to BMS-818251 to varying degrees when compared to wild type, with IC_50_ increasing by 85.0-fold (S375I), 2.5-fold (Q432T), 5.0-fold (A433V), 7.5-fold (M434I), and 47.5-fold (M475I).

**TABLE 1:** Neutralization results of BG505 pseudovirus Env mutants against BMS-818251^*a*^

BG505 env	IC_50_ (nM)	IC_50_ fold increase vs wild type	IC_80_ (nM)	IC_80_ fold increase vs wild type	DMS^*b*^ relative enrichment
Wild type	0.40	1.0-fold	1.00	1.0-fold	1.0-fold
W112L	>50.00	>125.0-fold	>50.00	>50.0-fold	5.6-fold
D113E	>50.00	>125.0-fold	>50.00	>50.0-fold	4.8-fold
S375I	34.00	85.0-fold	>50.00	>50.0-fold	3.3-fold
F382R	>50.00	>125.0-fold	>50.00	>50.0-fold	6.0-fold
M426L	>50.00	>125.0-fold	>50.00	>50.0-fold	5.2-fold
R429G	0.06	0.15-fold	0.17	0.17-fold	0.8-fold
Q432T	1.00	2.5-fold	4.00	4.0-fold	3.2-fold
A433V	2.00	5.0-fold	6.00	6.0-fold	3.3-fold
M434I	3.00	7.5-fold	10.00	10.0-fold	2.9-fold
M475I	19.00	47.5-fold	43.00	43.0-fold	4.5-fold

^*a*^ Pseudovirus neutralization assay has an expected variability of ±threefold (see details in Materials and Methods and the references therein). For comparison, we included DMS relative enrichment in the last column of this table. DMS relative enrichment is defined as the frequency of each amino acid mutation relative to wild type in the BMS-818251-treated vs untreated mock conditions.

^*b*^ DMS, deep mutational scanning.

One of the mechanisms of drug resistance is through the reduction of binding affinity of a drug to the target protein (11). To determine if reduction of binding affinity plays a role for the drug resistance observed here, we measured the binding affinity of recombinant HIV-1 Env mutants to BMS-818251 by isothermal titration calorimetry (Table 2). The F382R mutation completely abolished the binding of BMS-818251, explaining the complete resistance observed in the pseudovirus neutralization assay in Table 1. Similarly, M426L and S375I mutations decreased the binding affinity of BMS-818251 by 29.4-fold and 17.0-fold, respectively, corroborating the strong resistance observed in pseudovirus neutralization assay (Table 1). On the other end of the spectrum, Q432T and M434I only caused 1.6- and 2.8-fold reduction of binding affinity, respectively, consistent with the low resistance seen in the pseudovirus neutralization assay (Table 1). Three mutations that caused moderate reduction of binding affinity (6.2-fold, 7.8-fold, and 8.2-fold for M475I, W112L, and D113E, respectively) correlated with moderate to severe resistance in pseudovirus neutralization assay. The isothermal calorimetry results suggested that the reduction of binding affinity is likely the main mechanism leading to BMS-818251 resistance. A notable exception was observed in the correlation between binding affinity reduction and resistance in neutralization assays. The mutation R429G caused a reduction in binding affinity by 22-fold to BMS-818251 as measured by ITC; however, the neutralization assay showed that the R429G mutation led to a more sensitive phenotype compared to wild type, indicating that R429 might play an important role in the viral entry.

**TABLE 2:** Binding affinities of recombinant Env mutants to BMS-818251^*a*^

BG505 SOSIP gp140	*K*_*d*_ (nM)	*K*_*d*_ ratio	Δ*G* (kcal/mol)	Δ*H* (kcal/mol)	−TΔS (kcal/mol)	*N*
SOSIP wild type	5.2 ± 1.4	1	−11.8 ± 0.2	−7.9 ± 0.2	−3.9 ± 0.2	0.79 ± 0.05
W112L	39 ± 5	7.8	−10.5 ± 0.1	−4.3 ± 0.1	−6.2 ± 0.2	0.41 ± 0.05
D113E	41 ± 6	8.2	−10.5 ± 0.1	−3.8 ± 0.1	−6.7 ± 0.2	0.9 ± 0.1
V255F	23 ± 5	4.6	−10.8 ± 0.1	−4.0 ± 0.1	−6.8 ± 0.2	0.56 ± 0.05
S375I	85 ± 9	17	−10.0 ± 0.1	−3.8 ± 0.1	−6.2 ± 0.2	1.98 ± 0.05
F382R	>500	>100	–^*b*^	–	–	–
M426L	147 ± 24	29.4	−9.7 ± 0.1	−4.9 ± 0.2	−4.8 ± 0.2	0.81 ± 0.05
R429G	110 ± 18	22	−9.9 ± 0.1	−4.5 ± 0.1	−5.4 ± 0.2	1.1 ± 0.1
Q432T	8 ± 1	1.6	−11.5 ± 0.1	−7.7 ± 0.3	−3.8 ± 0.2	1.05 ± 0.05
A433V	65 ± 9	13	−10.2 ± 0.1	−6.2 ± 0.2	−4.0 ± 0.2	0.77 ± 0.05
M434I	14 ± 2	2.8	−11.1 ± 0.1	−3.7 ± 0.1	−7.4 ± 0.3	0.83 ± 0.05
M475I	31 ± 7	6.2	−10.7 ± 0.1	−2.1 ± 0.1	−8.6 ± 0.2	0.9 ± 0.1
BG505 DS-SOSIP gp140	19 ± 5	N/A^*c*^	−10.9 ± 0.1	−7.8 ± 0.2	−3.1 ± 0.1	1.0 ± 0.1

^*a*^ Isothermal titration calorimetry (ITC) was used to measure the binding activities between BMS-818251 and HIV-1 envelope protein mutants. Dissociation constant (*K*_*d*_), Gibbs free energy (Δ*G*), enthalpy (Δ*H*), entropy (-TΔ*S*), and stoichiometry (*N*) derived from ITC experiments are displayed (see Materials and Methods for details). *K*_*d*_ ratio is the ratio between mutant *K*_*d*_ and wild-type envelope proteins. SOSIP gp140 and DS-SOSIP gp140 are engineered recombinant HIV-1 envelope proteins that retained a closed prefusion conformation.

^*b*^ –, Binding affinity too low to reliably derive the ΔG, ΔH, −TΔS and N components.

^*c*^ N/A, Not applicable because of BG505 DS-SOSIP gp-140 is a different base construct.

**Fig 2:**
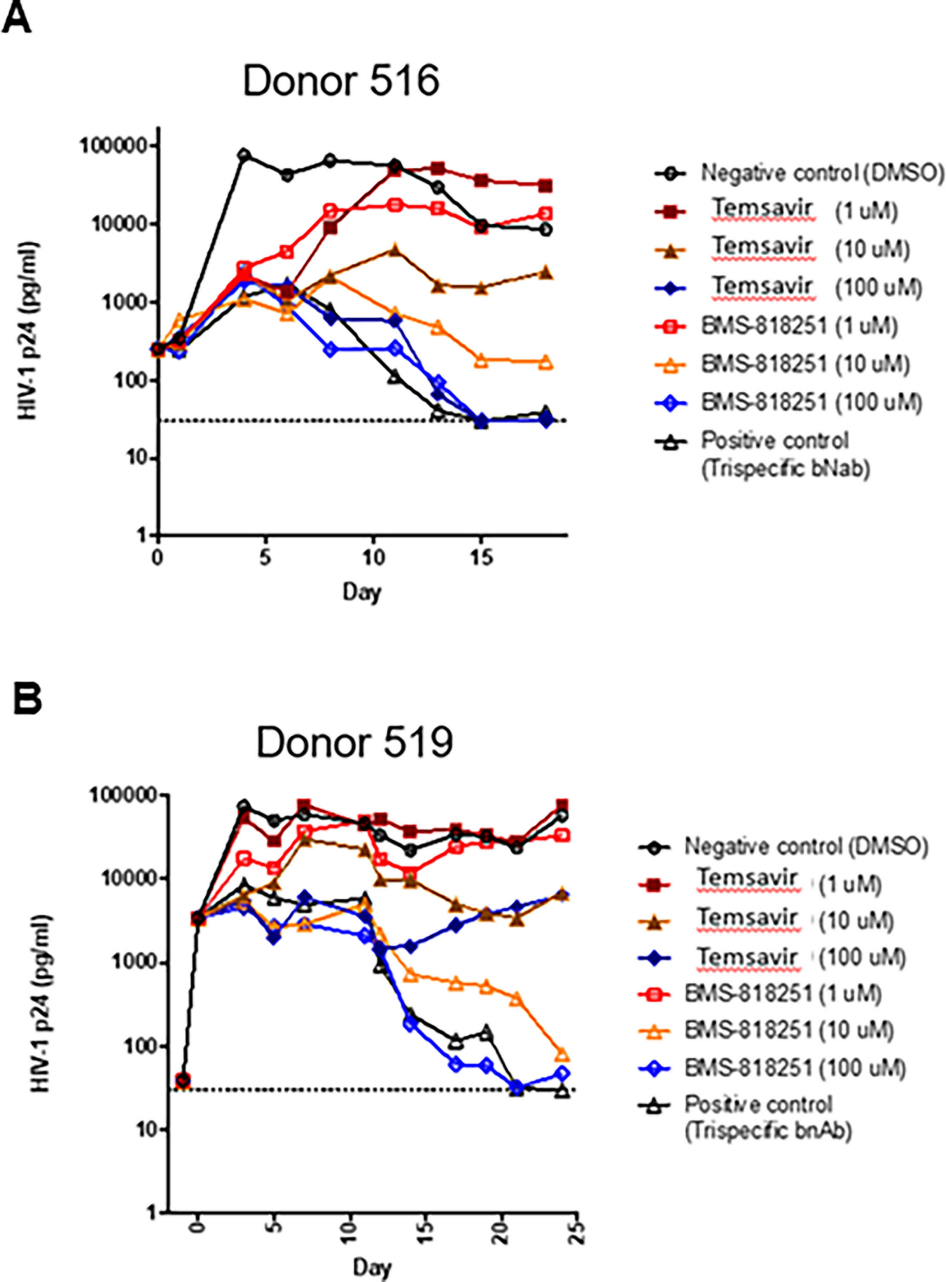
*Ex vivo* resistance assay using HIV+ CD4 T cells from two donors, 516 (A) and 519 (B), showed that BMS-818251 is more effective in suppressing viral growth than BMS-626529 (temsavir) when cultured with CD4 T cells from healthy donors. BMS-626529 (temsavir, the active compound of fostemsavir) was represented by solid symbols, and BMS-818251 was represented by empty symbols. For the ease of comparison, the two compounds at the same concentration are color-matched (red, 1 µM; orange, 10 µM; blue, 100 µM). Each data point represents a triplicate of p24 ELISA.

To better understand the potential effects of BMS-818251 in humans, we carried out *ex vivo* virus inhibition assays in cells derived from people living with HIV (PLWH). CD4^+^ T cells from two individuals living with HIV were activated and co-cultured with uninfected CD4^+^ T cells from healthy donors in the presence of either fostemsavir or BMS-818251, and virus growth was monitored for 18–25 days by HIV p24 enzyme-linked immunosorbent assay (ELISA) (Fig. 2). In cells from donor 516, both temsavir (the active compound of fostemsavir) and BMS-818251 achieved full suppression at the highest concentration (100 µM) tested. However, at lower concentrations (10 and 1 µM), BMS-818251 achieved better viral suppression than temsavir by roughly one order of magnitude. In cells from a second donor, donor 519, temsavir failed to suppress viral growth even at the highest concentration tested, while BMS-818251 could achieve suppression at both 100 and 10 µM concentrations. Viral growth was initially suppressed by 1 µM BMS-818251 treatment, followed by a rebound, suggesting enrichment of preexisting resistance mutations. However, despite the evidence of preexisting resistance in donor 519, full suppression was achieved with 10 and 100 µM of BMS-818251 by day 25. These *ex vivo* viral growth suppression data were consistent with the previously published pseudovirus neutralization assays, where BMS-818251 exhibited higher potency than temsavir (9).

**Fig 3:**
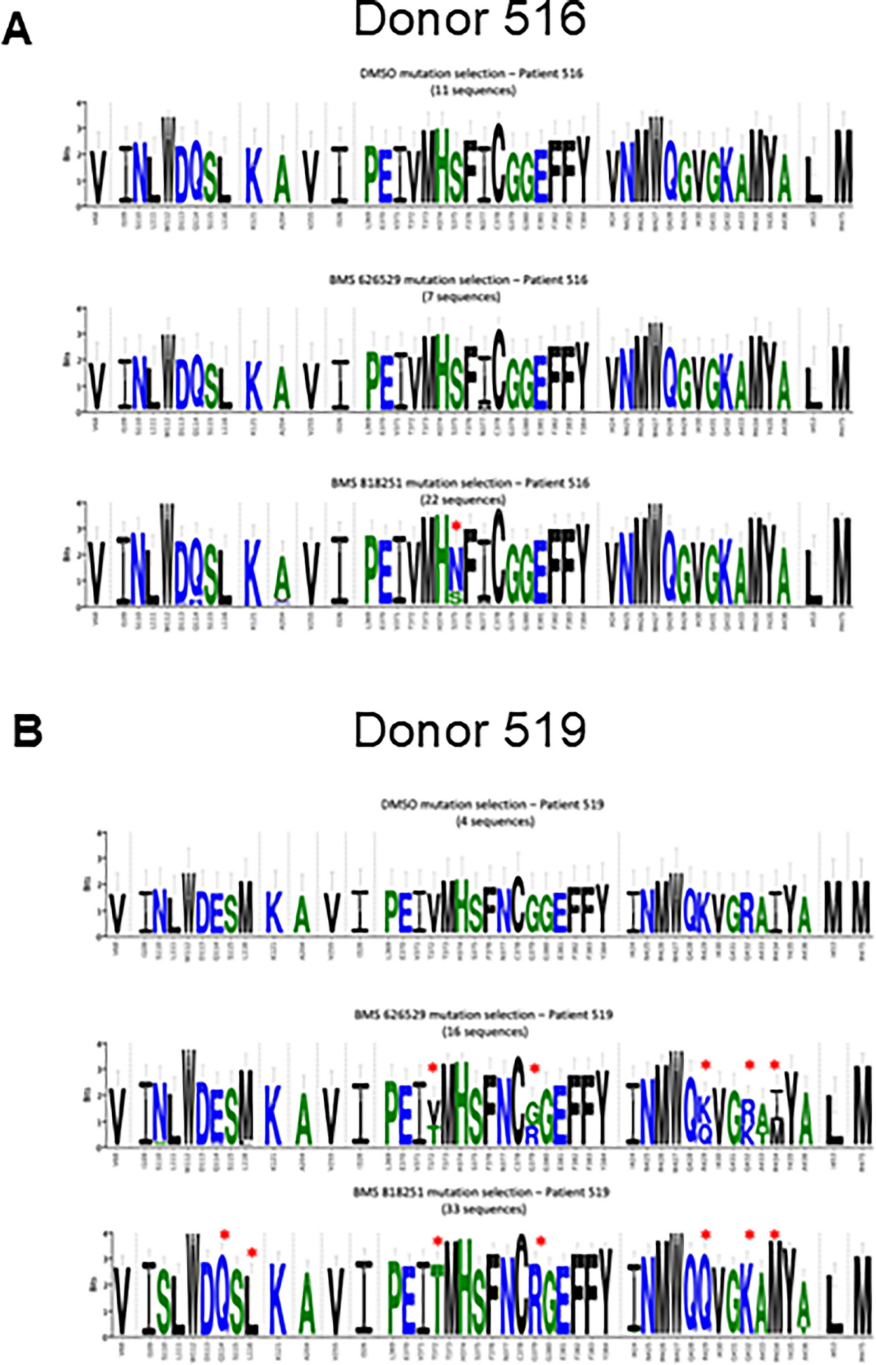
Sequencing of Env from treated cells reveals sites of selection that differ between BMS-818251 and temsavir. Env sequences recovered from treated T cells of donor 516 (**A**) and donor 519 (**B**) were compared within each donor to determine potential escape mutations. Enriched mutations are highlighted by asterisks. In cells from patient 516, only one mutation at position 375 was enriched in the BMS-818251-treated group (highlighted by a red asterisk in lower left plot). In contrast, in patient 519, five mutations at positions 372, 379, 429, 432, and 434 were enriched in groups treated by temsavir (BMS-626529, middle right plot) and BMS-818251 (lower right plot). Additional mutations at positions 114 and 116 were uniquely enriched in the BMS-818251-treated group in cells from patient 519. Logo plots created at weblogo.com. The *x*-axis shows amino acids and positions based on HxBc2 standard. The height of the entire stack of residues at a specific position represents the information content in bits; error bars show an approximate Bayesian 95% confidence interval.

DNA sequences of Env recovered from the suppression assay cultures were sequenced to identify potential escape mutations in an *ex vivo* setting (Fig. 3). In cells from donor 516, only one mutation, S375N, was enriched in the BMS-818251-treated group. In the deep mutational scanning (Fig. 1), an asparagine (N) mutation is among the most enriched escape mutations at site S375 and across Env. This suggests that the *in vitro* deep mutational scanning method can effectively predict *ex vivo* escape mutations. In cells from donor 519, five mutations at positions 372, 379, 429, 432, and 434 were enriched in groups treated with temsavir and BMS-818251. Positions 372 and 379 are close to position 375, where S375N enrichment was observed in donor 516 treated with BMS-818251. Deep mutational scanning was able to detect residues 372 and 379 as potential escape hotspots, albeit at a lower frequency than residue 375. However, unlike residue 375, residues 372 and 379 are not in direct contact with the inhibitor in the co-crystal structure (Fig. 1C). Mutations at 372 and 379 thus likely affect drug binding indirectly, possibly by altering the local environment and conformations of nearby residues (including residue 375). Of the other three common enriched mutations at positions 429, 432, and 434, residues 429 and 432 form direct contacts with the “tail” functional group of BMS-818251 (Fig. S2) through polar interactions in the co-crystal structure. The enriched mutations at positions 429 and 432 essentially reverted the charges of the side chains and potentially disrupted the binding of the BMS-818251 compound. As seen in Tables 1 and 2, mutation R429G significantly reduced the neutralization of IC_50_ and binding affinity of BMS-818251. A glutamine mutation at this position can potentially have a more negative effect than a glycine mutation in terms of neutralization and binding affinity. Finally, we observed that in the donor 519 sample treated with BMS-818251, there were two unique mutations at positions 114 and 116. These two mutations represented reversion back to wild-type Q114 and L116 from the initial donor virus sequences. In donor 516, these two positions were Q114 and L116 in the dimethyl sulfoxide (DMSO)-treated group as well as the two groups treated by either temsavir or BMS-818251. Hence, it is unlikely that these two mutations were selected to escape BMS-818251 treatment in donor 519.

## DISCUSSION

In this study, we demonstrate that BMS-818251 has significantly higher potency inhibiting viral growth from CD4^+^ T cells derived from people living with HIV *ex vivo* when compared to fostemsavir. Fostemsavir, the first-in-class HIV-1 attachment inhibitor, demonstrated efficacy in treating multidrug-resistant HIV-1 patients, but resistance mutations have been observed in clinical trials, prompting the development of improved next-gen analogs. BMS-818251 maintains a resistance profile largely overlapping fostemsavir while displaying increased potency across a broad range of HIV-1 strains. The higher affinity of BMS-818251 for the HIV envelope protein, primarily due to its additional tail functional group, likely contributes to its improved activity. This suggests that modifications enhancing drug-binding affinity can yield substantial therapeutic benefits.

Compared to other small-molecule entry inhibitors, such as maraviroc (a CCR5 antagonist), BMS-818251 targets a distinct step in the viral entry process. While maraviroc resistance arises due to tropism shifts or mutations in the CCR5-binding region of gp120, fostemsavir and BMS-818251 resistance mutations are confined to the gp120 drug-binding pocket. Our findings suggest that the escape mutations mapped for BMS-818251 likely are not cross-resistant with maraviroc or other entry inhibitors, supporting its potential use in combination therapy to prevent resistance development. Future studies should investigate how BMS-818251 interacts with other entry inhibitors and whether sequential or combined regimens could further extend treatment durability.

A deep mutational scanning approach could be valuable for monitoring resistance mutations both during clinical trials and after regulatory approval for human use. It allowed us to map resistance mutations at single-amino acid resolution, revealing a clustering of enriched mutations around the BMS-818251 binding site. Notably, the S375I/N, M426L, and M475I mutations, previously observed in fostemsavir-treated patients, were also enriched in our study, underscoring their clinical relevance. This suggests that these residues serve as common resistance hotspots for this class of inhibitors.

Eleven mutants in deep mutational scanning were selected for further characterization by pseudovirus neutralization, which verified and quantified the extent of resistance. Reduction of binding affinity, associated with resistance, to BMS-818251 for each of the mutations was determined by ITC. While most escape mutations reduced BMS-818251 binding affinity (ITC experiments) with accompanying increased resistance (pseudovirus neutralization assays), a notable exception was R429G, which displayed an increase in susceptibility to BMS-818251 despite significantly lower binding affinity (reduced by ~22-fold) to the inhibitor. This suggests that R429 plays a critical role in viral entry, and its alteration might destabilize the functional conformation of the envelope protein, making the virus more vulnerable to inhibition. Further structural studies are needed to clarify whether similar destabilizing mutations could be exploited for therapeutic advantage.

In a previously determined complex structure of BMS-818251 and the HIV-1 envelope protein, we have proposed that the high potency of BMS-818251 is due to additional interaction between the drug and the envelope protein. A tail functional group of BMS-818251, which fostemsavir lacks, established additional interactions with R429 and Q432 of the envelope protein, leading to higher binding affinity and higher potency. Interestingly, some resistance mutations were observed for the amino acid Q432, corroborating the role Q432 played in interacting with BMS-818251. However, in the deep mutational screening experiment, very low mutation enrichment was observed for R429, suggesting R429 may be critical to the fusion process.

A notable exception among the resistance mutations was F382R, which appeared to completely abolish binding to BMS-818251 in the ITC assay. Upon closer examination, quantitative analysis of SDS-PAGE (Fig. S3) revealed that the recombinant F382R Env protein underwent cleavage into two distinct fragments of approximately 62 and 17  kDa, consistent with proteolytic processing of the gp120 subunit. This pattern suggests that the arginine substitution at position 382 may have introduced a novel protease cleavage site, as arginine residues are frequently found at protease recognition motifs. Despite this cleavage, the F382R mutant retained membrane fusion capacity in the pseudovirus assay and was among the most highly enriched variants in the deep mutational scanning experiment, indicating that the mutation does not grossly impair viral fitness in its native trimeric form. However, the altered structural integrity likely perturbed the BMS-818251 binding site, thereby explaining the lack of detectable binding in the ITC assay. This case underscores the importance of verifying protein integrity when interpreting biophysical binding data and highlights the complexity of correlating *in vitro* binding with functional escape.

The *ex vivo* assays provided strong evidence that BMS-818251 achieves superior viral suppression compared to fostemsavir, even in donor samples harboring preexisting resistance mutations. In donor 516, BMS-818251 consistently exhibited greater potency at lower concentrations, suggesting a higher barrier to resistance emergence. In donor 519, viral rebound was observed with 1 µM BMS-818251, but complete suppression was achieved at 10 and 100 µM concentrations. This highlights the importance of dosing strategies in preventing resistance selection.

Interestingly, the sequencing of Env from *ex vivo* treated cells revealed an overlap between escape mutations observed *in vitro* and those emerging under drug pressure in a physiological setting. For instance, the S375N mutation—one of the most enriched in the deep mutational scanning—was the only mutation selected in donor 516, reinforcing the predictive value of our *in vitro* resistance profiling. In contrast, donor 519 exhibited a broader range of mutations, including changes at residues 372, 379, 429, 432, and 434, suggesting a more complex resistance landscape. The variation in resistance pathways between donors raises important clinical questions, such as (i) do preexisting polymorphisms in HIV envelope protein influence the trajectory of resistance evolution? and (2) could patient-specific resistance profiles help tailor treatment regimens? Our findings suggest that resistance monitoring during treatment could inform personalized adjustments in therapy, maximizing antiviral efficacy while minimizing resistance risk.

In the deep mutational scanning assay (*in vitro*), positions 372 and 379 were identified as potential escape hotspots, but they showed lower enrichment compared to position 375 (Fig. 1). This suggests that while mutations at these sites can contribute to resistance, they may not be the primary escape mechanism under strong BMS-818251 selection pressure *in vitro*.

In the *ex vivo* virus suppression assay, mutations at positions 372 and 379 were enriched in donor 519 but not in donor 516 (Fig. 3). The emergence of these mutations in donor 519 suggests that preexisting viral diversity or selective pressures in the donor’s viral population influenced escape pathways differently in the *ex vivo* setting. Unlike residue 375, which directly interacts with the inhibitor, positions 372 and 379 are not in direct contact with BMS-818251 in the co-crystal structure. Their role in resistance is likely indirect, possibly altering the local conformational environment of neighboring residues, including 375, which may reduce drug-binding efficiency.

Thus, while both assays detected changes at positions 372 and 379, the deep mutational scan identified them as minor escape sites, whereas the *ex vivo* virus suppression assay showed stronger selection at these sites in donor 519. This suggests that while *in vitro* screening captures broad resistance potential, *in vivo* or *ex vivo* conditions may introduce additional selective pressures that favor alternative resistance pathways.

### Conclusion

BMS-818251 represents a promising improvement over fostemsavir, exhibiting higher potency, a well-defined resistance profile, and superior *ex vivo* viral suppression. The comprehensive resistance mapping presented in this study lays the foundation for future drug design efforts, emphasizing the importance of optimizing binding affinity and identifying structural vulnerabilities in HIV-1 gp120. BMS-818251 has the potential to serve as an effective treatment option for patients with multidrug-resistant HIV-1, warranting further clinical evaluation.

## MATERIALS AND METHODS

### Generation of mutant virus libraries

We have previously described the BG505.T332N mutant proviral DNA libraries and the resulting functional mutant virus libraries (12). In brief, triplicate mutant BG505.W6M.C2.T332N env libraries that contained randomized codon-level mutations to sites 31-702 (HXB2 numbering is used here and throughout this article) were independently generated and cloned into Q23.BsmBI.ΔEnv proviral plasmid (12). These proviral plasmid libraries, as well as wild-type proviral plasmid, were transfected into 293T cells (ATCC). We then passaged the transfection supernatant at an MOI of 0.01 TZM-bl infectious units per cell in SupT1.CCR5 cells. The resulting genotype-phenotype linked mutant virus libraries were concentrated via ultracentrifugation.

### Mutational antigenic profiling

To identify comprehensively potential BMS-818251 resistance mutations, we incubated the mutant virus libraries with and without BMS-818251, infected SupT1.CCR5 cells, and subsequently identified the mutant viruses that were enriched upon drug selection using deep sequencing. This approach is similar to the mutational antigenic profiling process we have previously used to map antibody (10) and fusion inhibitor (13) escape. Briefly, 1 × 10^6^ infectious units of three mutant virus libraries were incubated in the presence of 12.5, 15.0, or 20.0 nM of BMS-818251 for 1 hour and then infected into 1 × 10^6^ SupT1.CCR5 cells in R10 (Roswell Park Memorial Institute [RPMI] media with 10% fetal bovine serum (FBS), 1% 200 mM L-glutamine, and 100 units/mL of penicillin and streptomycin), containing 100 µg/mL diethylaminoethyl (DEAE)-dextran. Three hours post-infection, cells were resuspended in 1 mL R10 containing no DEAE-dextran. Twelve hours post-infection, non-integrated viral cDNA was isolated using a miniprep. As mock-selected controls, each mutant virus library was infected into cells without BMS-818251 selection. Selected and mock-selected viral cDNA was then sequenced with a barcoded subamplicon sequencing approach as described below, which introduces unique molecular identifiers used to correct sequencing errors.

We used dms_tools2 (https://jbloomlab.github.io/dms_tools2/) (14) to analyze the deep mutational scanning sequencing data. Differential selection has been previously described (10) and is documented in more detail at https://jbloomlab.github.io/dms_tools2/diffsel.html. Briefly, the differential selection for each mutation is the logarithm of the mutation’s enrichment in the drug-selected mutant virus library relative to the non-selected control library. We present the median differential selection metrics of triplicate experiments throughout the article.

### HIV-1 pseudovirus neutralization assays

Neutralization was measured using single-round-of-infection HIV-1 Env-pseudoviruses and TZM-bl target cells (ARP cat no. #8129) described in detail in previous publications (15, 16). Pseudoviruses were prepared by co-transfection of Env expression plasmids with backbone plasmid pSG3dEnv in HEK293T cells and titered in TZM-bl cells as described (17). Mutagenesis of the Env plasmid was performed by GeneImmune USA (Rockville, MD, USA) and verified by Sanger sequencing. BMS-818251 compound was prepared at 400 µM with 4% DMSO in phosphate-buffered saline (PBS) and diluted 20 times as the starting concentration for serial dilution in the neutralization assays. Pseudoviruses of the HIV-1 BG505 strain (clade A) and its mutants were incubated with serial dilutions of drug compounds for 1 hour, then added to TZM-bl target cells, which have a luciferase reporter gene. After incubation at 37°C for 2 days, infection of target cells was measured by luciferase activity (17). Samples were run in duplicate, and experiments were performed one to three times.

Neutralization curves were fit by non-linear regression using a five-parameter hill slope equation (16). IC_50_ and IC_80_ values were derived from the dose-response curves. This assay has an expected variability of ±threefold (18). To provide additional context on assay variability and clarify how differences between wild-type and mutant pseudoviruses were interpreted, we generated two statistical plots presented in Fig. S4. In the first panel, IC_50_ values from seven independent wild-type BG505 pseudovirus assays (each performed in duplicate) are plotted alongside the values for each mutant. The geometric mean and ±threefold range of the wild-type IC_50_ are shown, highlighting which mutants fall outside this conservative threshold for meaningful change. The second panel presents the fold change in IC_50_ values relative to the wild type for each mutant, reinforcing the observation that all tested mutants exceed the ±threefold cutoff. Together, these plots support the conclusion that the resistance mutations characterized in this study exhibit biologically meaningful differences from the wild-type control.

### Protein expression and purification

BG505 SOSIP.664 is a construct with modifications, including truncation at amino acid residue 664 and stabilization mutations, that enabled the production of fully cleaved, prefusion-closed (native-like), soluble HIV-1 Env trimer (19). Protein samples were purified following published protocols (9) with modifications described below. For 1 L of Expi293 cell culture (Thermo Fisher Scientific, California, USA), 600 µg of BG505 SOSIP.664 (with N332 glycan) expression plasmid and 150 µg expression plasmid for human Furin protease were diluted in 50 mL Opti-MEM medium (Life Technology). Three milliliters of Turbo293 transfection reagent (Speed BioSystems) was diluted into 50 mL Opti-MEM medium and incubated for 5 min at room temperature. Diluted transfection reagent was then added to the equal volume of Opti-MEM containing plasmids and incubated for 15 min at room temperature. The transfection reagent and DNA complex (100 mL total) were then added into 800 mL of Expi293 cells at 2 million/mL. On the second day of transfection, 80 mL HyClone SFM4HEK293 booster medium was added to the cell cultures, which were then returned to shaker incubator at 120 rpm, 37°C, and 9% CO_2_ for additional 6 days.

Supernatant of cell culture was affinity-purified over 2G12 antibody column, eluted by 3 M MgCl_2_, followed by size exclusion chromatography in 5 mM HEPES buffer and 150 mM NaCl. The protein yield after size exclusion was ~1 mg from 1 L of cell culture, with high purity (except for F382R mutant, which was cleaved into smaller fragments). All assays requiring purified recombinant proteins were conducted on the same batch of purified protein. Mutant plasmids were created by site-directed mutagenesis carried out at GeneImmune USA, followed by Sanger sequencing to verify mutations. Mutant protein samples were produced by the same protocol as the wild type, yielding various amounts of purified protein.

### ITC experiment

The effect of resistance mutations in HIV-1 Env on the binding thermodynamics for BMS-818251 was studied by ITC using a MicroCal VP-ITC from Malvern Panalytical (Northampton, MA, USA). BMS-818251 was first dissolved in 100% DMSO at a concentration of 10 mM and then diluted into PBS with additional DMSO to the experimental concentration of 30 µM in the presence of 4% DMSO. Recombinant wild-type and mutant HIV-1 Env BG505 SOSIP protein was dialyzed into PBS, pH 7.4, and then further diluted to 1.5–1.8 µM (monomer basis) in PBS with 4% DMSO. Titrations were performed at 37°C by injecting 10 µL aliquots of the inhibitor solution into the calorimetric cell containing the Env protein (cell volume ~1.4 mL). The heat evolved upon each injection was obtained from the integral of the calorimetric signal, and the heat associated with binding was obtained after subtraction of the heat of dilution. The enthalpy change, Δ*H*, the association constant (*K_a_*) (the dissociation constant, *K_d_* = 1/*K_a_*) and the stoichiometry, *N*, were obtained by non-linear regression of the data. Gibbs free energy (Δ*G*) was calculated from the binding affinity using Δ*G* = *−*RTln*K_a_* (*R* = 1.987 cal/[K × mol], and *T* is the absolute temperature in kelvin). The entropy contribution to Gibbs energy, *−*TΔS, was calculated from the relation Δ*G* = Δ*H* −TΔS. Each titration is composed of a minimum of 28 points, which were to estimate statistical error. Each experiment was carried out twice.

### *Ex vivo* virus inhibition assay

The *ex vivo* virus inhibition assay was performed as described previously (20). In brief, CD4^+^ T cells were negatively selected from frozen peripheral blood mononuclear cell (PBMC) samples obtained from viremic PLWH using the CD4^+^ T-cell isolation kit (Miltenyi Biotec) according to manufacturer’s indicated procedure. Enriched CD4^+^ T cells were activated in complete Roswell Park Memorial Institute (cRPMI) media (RPMI media + 10% FBS + 1% Pen-Strep) containing interleukin (IL)-2 (10 U/mL, Sigma) and PHA-P (5 mg/mL, Sigma) overnight. CD4^+^ T cells from three different healthy donors were activated separately overnight using the same activation media. All cell cultures were counted and then combined into a single bulk culture at a ratio of 1:4, PLWH donor to uninfected CD4^+^ T cells, with equal parts from each of the three healthy donors. The bulk culture was maintained in cRPMI + IL-2 at a density of 1 million cells/mL for approximately 7 days until the HIV-1 gag (p24) level detected by antigen capture ELISA (ABL) in supernatant was above 100 pg/mL. Cells were then split and plated into a 96-well culture plate at 50,000 cells/200 µL/per well. Temsavir or BMS-818251 was diluted in cRPMI + IL-2 media and added to respective wells at 1, 10, or 100 µM concentration. DMSO was utilized as a negative control for viral replication. All conditions were plated in triplicate wells, and assays were maintained for approximately 4 weeks by adding 25,000 activated CD4^+^ T cells from healthy donors to each well once a week. One hundred microliters of supernatant was removed three times a week for p24 capture ELISA assay (ABL), developed according to the manufacturer’s protocol. Removed media were replaced with fresh media plus temsavir, BMS-818251, or DMSO at appropriate concentrations.

### Single-genome amplification and sequencing of HIV-1 Env

Virions from 50 µL of culture supernatants from the different viral resistance assays had RNA extracted utilizing the RNAdvance viral kit (Beckman Coulter) as previously described (21). Viral RNA was subjected to cDNA synthesis and used for viral single-genome amplification (SGA) and sequencing of HIV-1 Env as previously described (22). Briefly, 30 µL of extracted RNA was reverse transcribed to cDNA using the SuperScript III reverse transcription kit (Thermo Fisher Scientific) and a gene specific primer (3′Env-outR1: TTGCTACTTGTGATTGCTCCATGT) according to manufacturer’s protocol. SGA consisted of a nested PCR in which the first round of each PCR reaction used the Taq Platinum High Fidelity (Thermo Fisher Scientific), the outer primers 3′Env-outR1 and 5′Env-outF1 (TAGAGCCCTGGAAGCATCCAGGAAG), and 1 µL of diluted cDNA; the second round reactions, also using Taq Platinum High Fidelity, utilized the inner primers 3′Env-inR2 (GTCTCGAGATACTGCTCCCACCC) and 5′Env-inF2 (TTAGGCATCTCCTATGGCAGGAAGAAG) and 1 µL of first-round PCR. Limiting dilution of viral cDNA was performed in order to generate single-genome templates and amplification efficiency of 30% of total number of reactions as previously described (20, 23). The amplification cycle consisted of denaturation at 94°C for 2 min, 35 cycles (first round) or 40 cycles (second round) of 94°C for 15 seconds, 55°C for 30 seconds, and 68°C for 4 min and a final extension at 68°C for 10 min. Amplicons were directly sequenced with the Big Dye Terminator technology (Applied Biosystems) and resolved on an ABI 3730 automated genetic analyzer. Sequencing reactions utilized the following primers: For13 (GAGAAAGAGCAGAAGACAGTGG), Rev14 (ACCATGTTATTTTTCCACATGTTAAA), For15 (CAGCACAGTACAATGTACACATGGAA), Rev15 (CTGCCATTTAACAGCAGTTGAGTTGA), For17 (AGCAGCAGGAAGCACTATGGGCGC), Rev17 (CCTGGAGCTGTTTAATGCCCCAGAC), For18 (CATATCAAATTGGCTGTGGTATAT), Rev18 (GGTGAGTATCCCTGCCTAACTCTAT), and For19 (GGAACCTGTGCCTCTTCAGCTACC). The full-length Env sequences were assembled and edited using Geneious Prime software 2022.0.1. Chromatograms with background, double peaks, or incomplete sequences were excluded from analysis. Amino acid frequencies of Env protein sequences were assessed by Logo plot analysis generated with the WebLogo 3 online tool (https://weblogo.threeplusone.com/).

### Human subjects

PBMCs were obtained from viremic PLWH donors enrolled in the VRC 601 trial (24). VRC 601 was a single-site, phase 1, open-label dose escalation study examining the safety and pharmacokinetics of the human mAb VRC-HIVMAB060-00-AB (VRC01) in HIV-infected adults. The study was conducted at the National Institutes of Health (NIH) Clinical Center by the VRC Clinical Trials Program, National Institute of Allergy and Infectious Diseases (NIAID), NIH (ClinicalTrials.gov NCT01950325).

## Data Availability

Nucleotide sequences of HIV-1 envelop protein mutants used for BMS-818251 binding experiments were deposited in the National Center for Biotechnology Information GenBank with the following accession numbers: PV904167, PV904168, PV904169, PV904170, PV904171, PV904172, PV904173, PV904174, PV904175, PV904176, PV904177, and PV904178.

## SUPPLEMENTAL MATERIAL

Supplemental File S1 aac.01910-24-s0001.csv All mutation differential selection values (median across replicates).

Supplemental Figures aac.01910-24-s0002.pdf Figures S1 to S4.

ASM does not own the copyrights to Supplemental Material that may be linked to, or accessed through, an article. The authors have granted ASM a non-exclusive, world-wide license to publish the Supplemental Material files. Please contact the corresponding author directly for reuse.
