# Supplementary material for: Fostemsavir analog BMS-818251 has enhanced viral neutralization potency and similar escape mutation profile
Source: Antimicrob Agents Chemother. 2025 Aug 27;69(10):e01910-24. doi: 10.1128/aac.01910-24 (PMC12486810; doi:10.1128/aac.01910-24)
Supplement: Supplemental Figures — Figures S1 to S4. [file aac.01910-24-s0002.pdf]

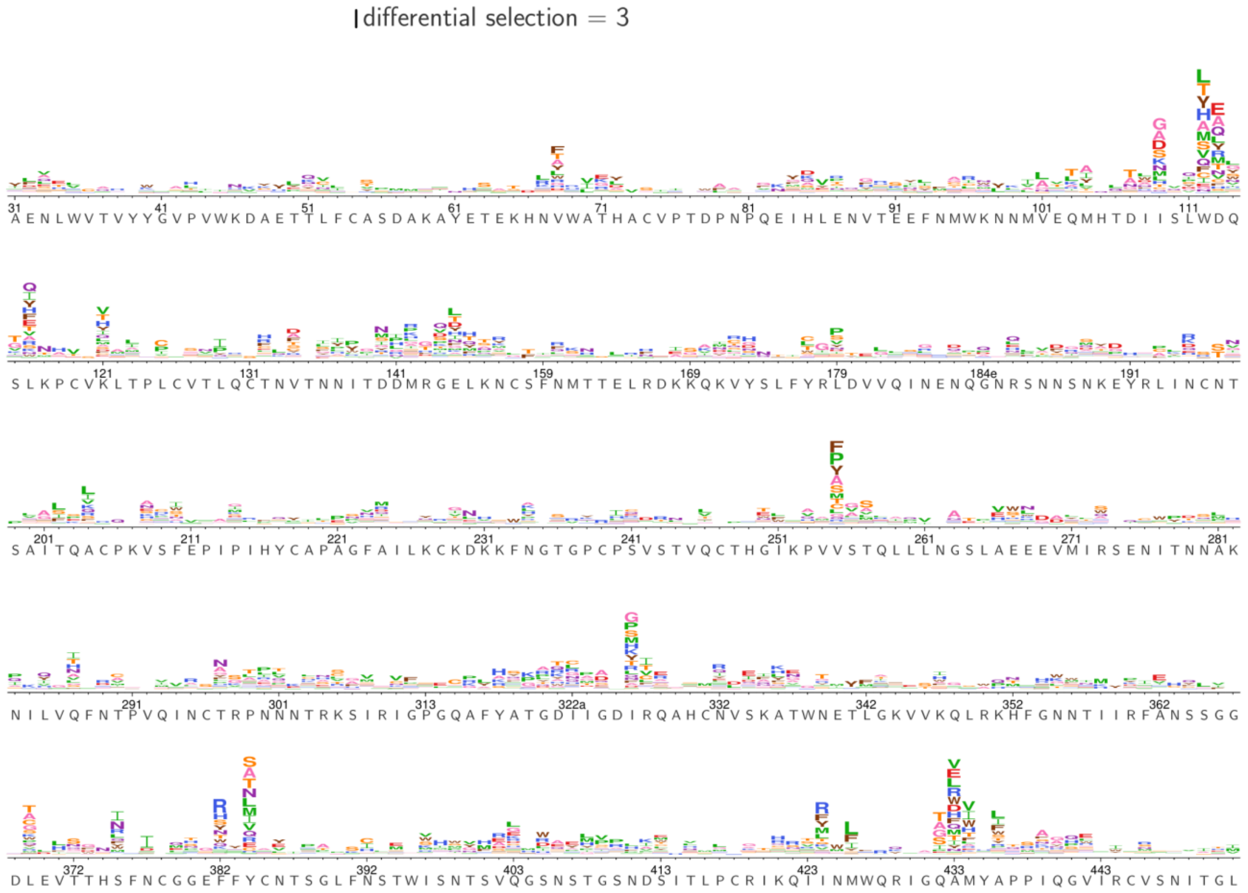

Figure S1. The complete mutation-level resistance profile across the length of the mutagenized portion of BG505 Env, similar to as plotted for a subset of sites in Figure 1B (median across replicates). All mutations that were enriched in BMS-818251 treated samples compared to samples with mock treatment were shown. Above the logoplot a perpendicular scale bar was shown next to the label “differential selection = 3”. Letters in the logoplot with the height of the perpendicular scale indicates they have a differential selection = 3, which means they have 2^3 = 8 fold of relative enrichment.

BMS-626529 (Temsavir) BMS-818251

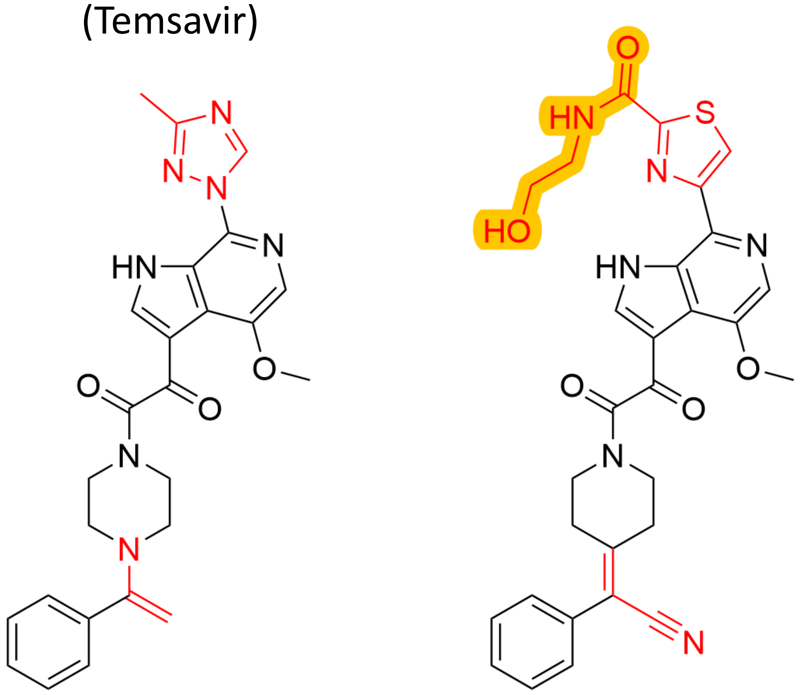

Figure S2. Structural comparison of temsavir and BMS-818251. Functional groups that are different between these two compounds are colored in red. The tail functional group is highlighted in orange shade.

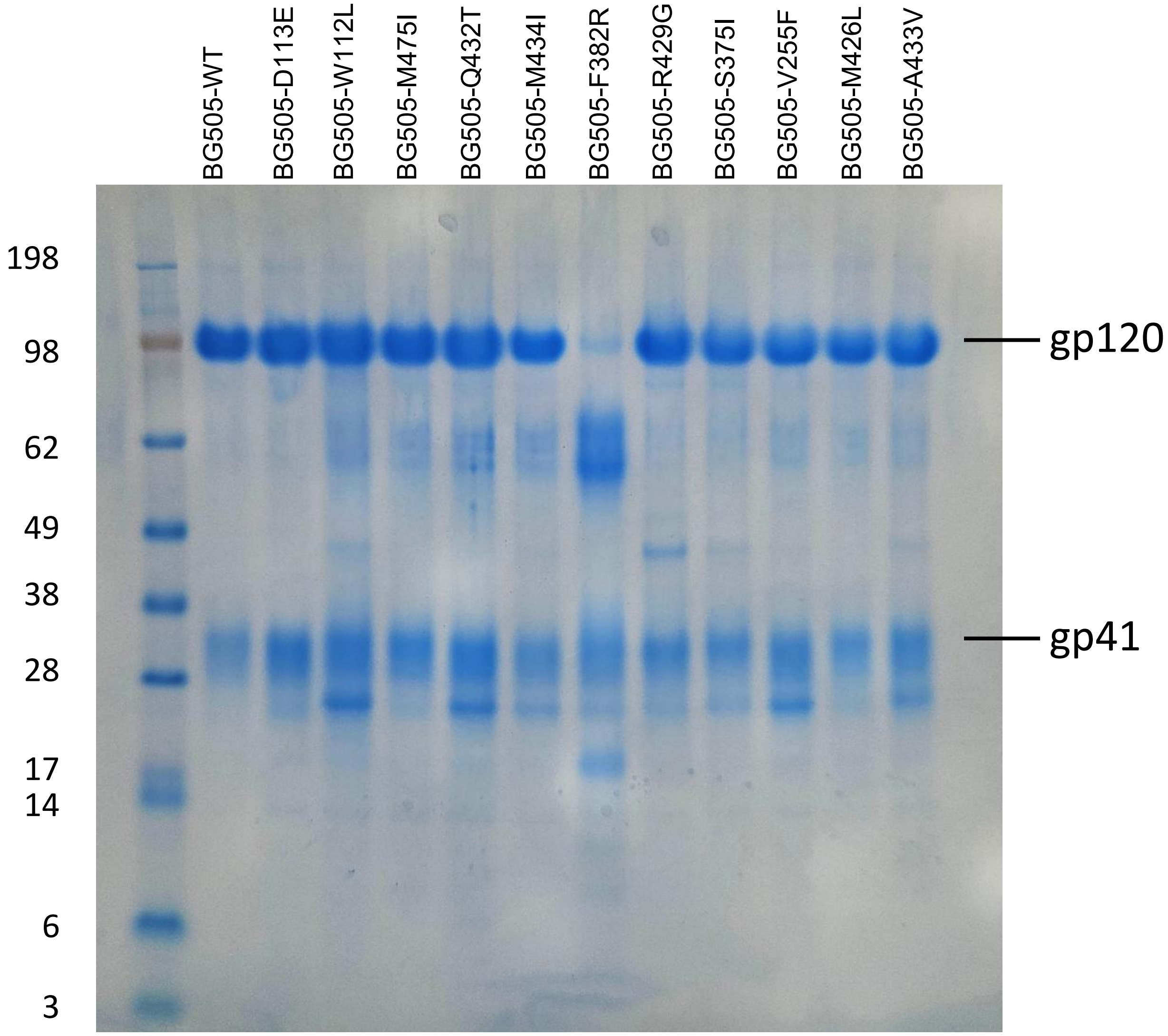

Figure S3. SDS-PAGE under reducing condition showed high purities of the recombinant BG505.SOSIP.664 and mutants used in this study. Note the F382R mutation led to unexpected cleavage of gp120 which explained its lack of binding to BMS-818251. These samples were stored at -80C for more than 3 years after ITC experiments were carried out. SDS-PAGE result demonstrated high stability of these recombinant proteins after long-term storage.

A

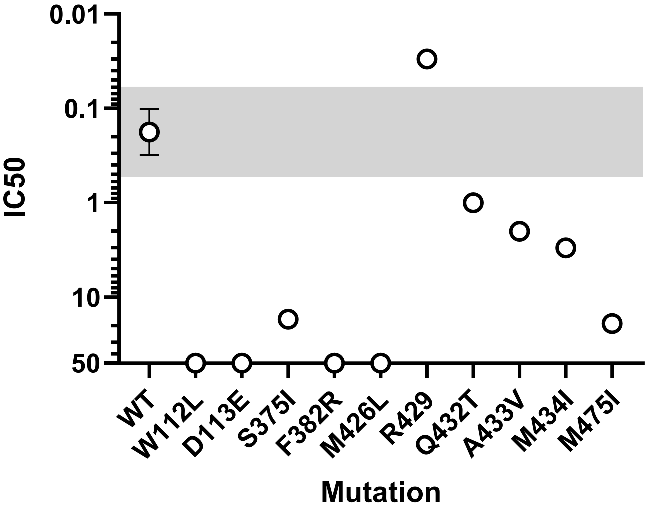

Error bars: geomean and geometric SD of 7 repeats, for wild type.
All mutants were run in one experiment, in duplcate.
Gray bar: 3-fold above or below geometric mean for wild type.

B

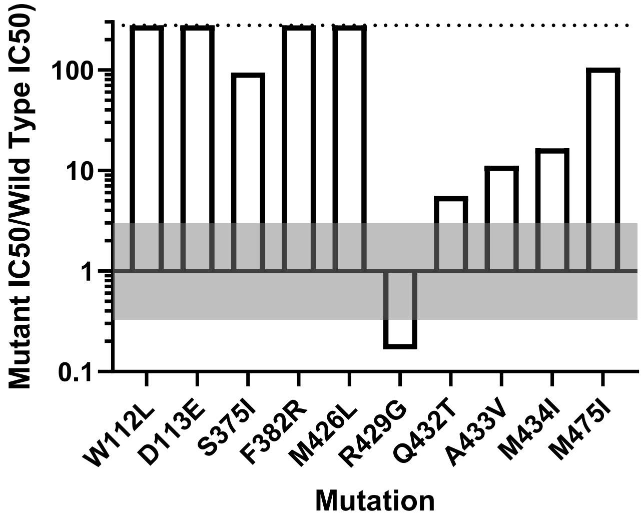

Gray bar: 3-fold above or below geometric mean of 7 experiments for wild type.
All mutants were run in one experiment, in duplicate.

Figure S4. Visualization of the 3-fold difference cut-off in pseudovirus neutralization assays. (A) IC_{50} values for each mutant alongside the geometric mean and geometric standard deviation of the wildtype. A shaded region corresponding to ±3-fold of the wildtype geometric mean is also indicated. Mutants Q432T, A433V, and M434I are closest to this threshold but still exceed it. (B) Fold-changes of IC_{50} between wildtype and mutants are plotted as an alternative representation. All reported mutants showed >3-fold increases in IC_{50}, which we consider to represent meaningful differences based on our established assay variability and prior publication precedent.
